# Photoelectrical Properties Investigated on Individual Si Nanowires and Their Size Dependence

**DOI:** 10.1186/s11671-021-03487-1

**Published:** 2021-01-28

**Authors:** Xiaofeng Hu, Shujie Li, Zuimin Jiang, Xinju Yang

**Affiliations:** 1 State Key Laboratory of Surface Physics, Fudan University, Shanghai, 200433 China; 2 Kunming Institute of Physics, Kunming, 650223 China

**Keywords:** Si nanowires, Photoconductive atomic force microscopy, Electrostatic force microscopy, Photoconductive property, Size-dependence

## Abstract

Periodically ordered arrays of vertically aligned Si nanowires (Si NWs) are successfully fabricated with controllable diameters and lengths. Their photoconductive properties are investigated by photoconductive atomic force microscopy (PCAFM) on individual nanowires. The results show that the photocurrent of Si NWs increases significantly with the laser intensity, indicating that Si NWs have good photoconductance and photoresponse capability. This photoenhanced conductance can be attributed to the photoinduced Schottky barrier change, confirmed by I–V curve analyses. On the other hand, electrostatic force microscopy (EFM) results indicate that a large number of photogenerated charges are trapped in Si NWs under laser irradiation, leading to the lowering of barrier height. Moreover, the size dependence of photoconductive properties is studied on Si NWs with different diameters and lengths. It is found that the increasing magnitude of photocurrent with laser intensity is greatly relevant to the nanowires’ diameter and length. Si NWs with smaller diameters and shorter lengths display better photoconductive properties, which agrees well with the size-dependent barrier height variation induced by photogenerated charges. With optimized diameter and length, great photoelectrical properties are achieved on Si NWs. Overall, in this study the photoelectrical properties of individual Si NWs are systematically investigated by PCAFM and EFM, providing important information for the optimization of nanostructures for practical applications.

## Introduction

Silicon nanowires (Si NWs) have attracted great attention in recent years due to their unique properties and compatibility with traditional silicon technology. Si NWs have been demonstrated for a variety of applications, such as integrated logic circuits, solar cells, thermoelectric devices, and biosensors [1–5]. Particularly, when arranged in a highly ordered way, Si NWs can greatly improve the light absorption and charge collection, making them possible to achieve high efficiency in both solar cells and photodetectors [6–8]. In the past decades, the controllable growth of such ordered nanowire arrays as well as the optimal fabrication of photovoltaic (PV) devices have been intensively investigated [9–11]. Conversely, there are much fewer fundamental studies about the photoelectrical characteristics on such Si NWs arrays, especially on individual nanowires inside the arrays.

In order to realize the applications of ordered nanowire arrays in solar cells and PV devices, it is extremely important to get a good understanding of their photoconductive properties. Nowadays, the photoconductive properties of nanowire arrays are generally investigated by macroscopic methods with the deposition of two-side electrodes under light irradiation [12, 13]. However, for more accurate analysis, it is necessary to achieve the properties on single or individual nanowires rather than averaged results. Besides the studies applying single nanowire devices which is not easy to fabricate, scanning probe microscopy (SPM)-based electrical measurements have revealed themselves as powerful techniques for electrical characterizations at nanoscale [14, 15]. Among these SPM techniques, conductive atomic force microscopy (CAFM) has been most often applied to study the conductive properties of individual nanostructures such as films, heterostructures as well as nanowires [16–20]. By combining with laser irradiation, it can be modified as photoconductive atomic force microscopy (PCAFM) which provides a route to investigate the photoconductive properties on individual nanostructures [21, 22]. In recent years, PCAFM has already been employed to photocurrent measurements on organic [23–26] and inorganic solar cells [27–29], as well as on some nanostructures, including microcrystalline Si thin films, CdS heterostructures, MoS_2_ films and ZnO NWs [30–33]. Yet, most of these studies focused on the influence of laser irradiation with varied power intensities or wavelengths, while a few researches concerned with the effect of nanowires’ size.

On the other hand, to achieve Si NWs array with excellent photoconductive properties, it is quite necessary to obtain their size dependence for the optimization of nanowires’ diameter and length. Hence in recent decades, many efforts have been devoted to revealing the size dependence of photoconductive properties by using macroscopic methods or single nanowire devices [34, 35]. In the aspect of length dependence, many researches found that the photocurrent increased with the increasing of nanowire length below a specific value varied from 1 to 18 μm and then decreased as the length further increased [12, 36, 37], while another study reported that the photoconductance increases sublinearly with decreased length [38]. Meanwhile, the results of diameter dependence were yet much inconsistent. For example, the work by Kim et al. found that photoconductance of intrinsic Ge nanowires increased with the decreased diameter [35], while other works on GaN nanowires found the photocurrent increased as the diameter increased [39]. Therefore, the size dependence of photoconductive properties on nanowires is far from reaching a good and common understanding.

In this paper, ordered arrays of vertically aligned Si NWs with controllable diameters and lengths are successfully fabricated by the method of nanosphere lithography (NSL) combined with metal-assisted chemical etching (MACE), as reported in previous studies [1, 40]. Their photoconductive properties are investigated by PCAFM without any further nanofabrication. Our results demonstrate that the photocurrent measured on individual Si NWs increases greatly with the laser intensity, and the increasing magnitude is obviously related to the nanowires’ size. Si NWs with smaller diameters and shorter lengths are more photoconductive. On the other hand, the measurements performed by electrostatic force microscopy (EFM) combined with laser irradiation provided the information of photogenerated charges and barrier height modification, which can be employed to explain the size-dependent photoenhanced conductance of Si NWs. Therefore, this study not only reveals the size-dependent photoelectrical properties of Si NWs but also suggests that PCAFM and EFM are effective tools in investigating the photoelectrical properties of individual nanostructures as well as to explore the size (or other parameters) dependence.

## Materials and Methods

### Materials

The Si wafers were purchased from MTI (China). Deionized water (DI, 18.2 MΩ cm) was obtained from an ultrafiltration system (Milli-Q, Millipore, Marlborough, MA). Acetone, methanol, sulphuric acid, hydrogen peroxide, and hydrofluoric acid were purchased from Sinopharm Chemical Reagent (China). The suspensions (2.5 wt% in water) of polystyrene spheres (PS, 490 nm in diameter) were purchased from Duke Scientific (USA).

### Fabrication and Characterization of Si NWs

**Fig. 1:**
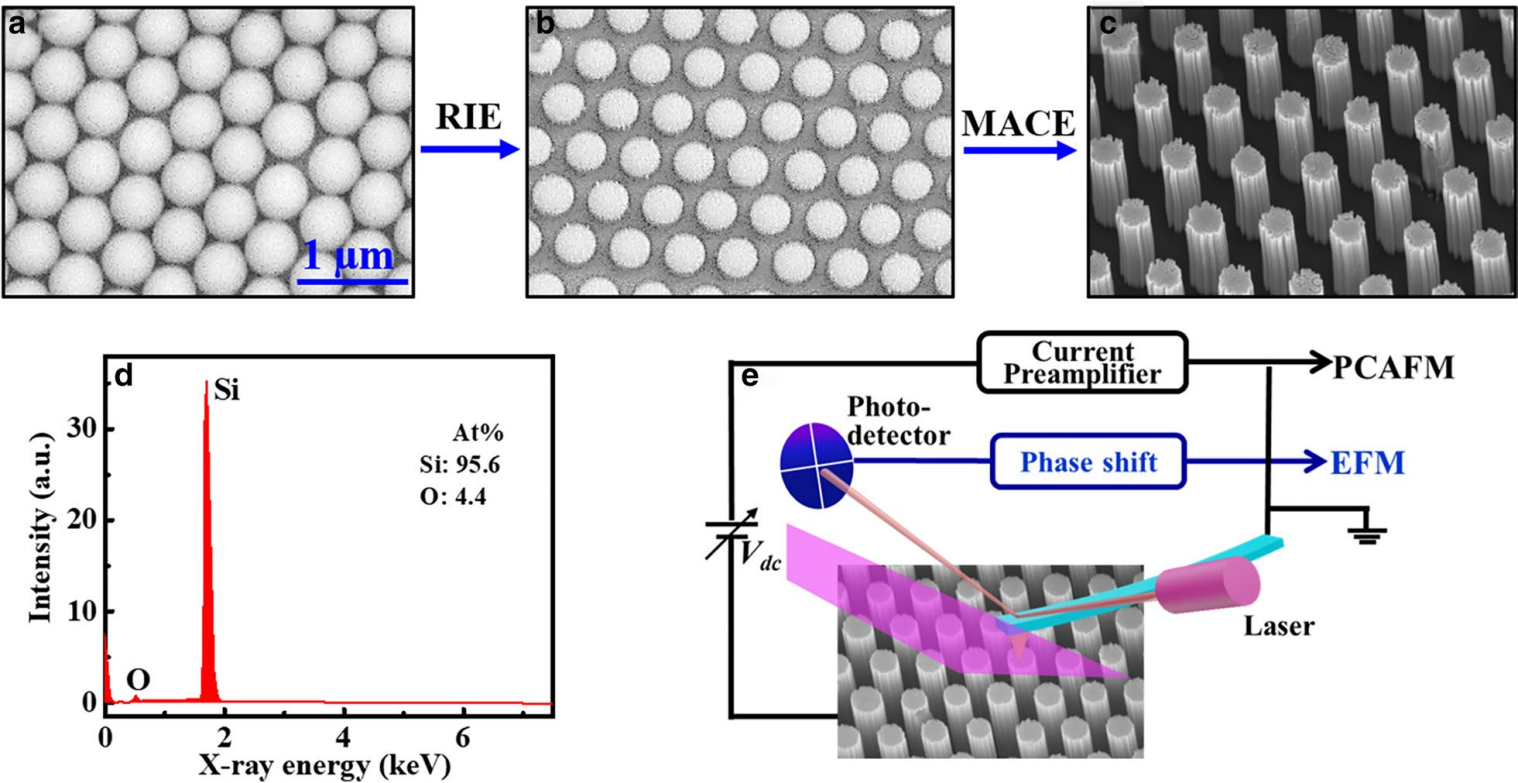
**a**–**c** SEM images of main procedures to fabricate vertically aligned Si NWs array: **a** Self-assembled PS monolayer, **b** diameter-reduced PS monolayer and **c** fabricated Si NWs array. **d** EDX spectrum measured on Si NWs. **e** Schematic diagrams of PCAFM and EFM under laser irradiation

Vertically ordered silicon nanowire arrays were fabricated by NSL and MACE, as reported in previous studies [1, 40]. The major fabrication processes are simply described as follows. Firstly, polystyrene spheres (PS) were self-assembled onto the chemically cleaned Si wafer (n-type, 0.01–0.02 Ω cm). Next, the diameter of PS spheres was reduced by reactive ion etching (RIE, Trion Technology) (50 W, 70 mTorr) to a desired value, and the diameter-reduced PS monolayer acted as a mask in the following procedures. After a 20 nm Au film deposition by ion sputtering which acted as a catalyst for the following MACE treatment, the sample was dipped in the mixed solution of HF (40%) and H_2_O_2_ (30%) with a volume ratio of 4:1 for MACE process and vertically aligned Si NWs were produced by this procedure. Finally, the remaining Au layer and PS spheres were removed by soaking the sample in KI/I_2_ mixed solution and tetrahydrofuran solution, respectively. The morphology after each step was checked by scanning electron microscopy (SEM, SIGMA300). Typical SEM images of original self-assembled PS monolayer, diameter-reduced PS monolayer, and the fabricated Si NWs after removing Au layer and PS spheres were shown in Fig. 1a–c, respectively. It can be seen that ordered arrays of vertically aligned Si NWs were achieved in a large scale. Moreover, by adjusting the RIE and MACE time, the nanowires’ diameter and length can be well controlled [40].

Besides, the composition of such nanowires was measured by using energy dispersive X-ray spectroscopy (EDX, OXFORD, Aztec X-Max 80). A typical EDX spectrum measured on Si nanowires after HF dipping is displayed in Fig. 1d. The results show that the nanowires are dominated by silicon (~ 95.6%) except traces of oxygen (4.4%). For confirmation, the EDX measurements were repeated for many times on different areas of the sample, and the measured results were well accordant, with the oxygen concentration varied from 0 to 7.2%. Therefore, it could be roughly considered that the fabricated Si NWs are pure and free of any other impurities, except a slight oxidation on the surface. Our results agree well with those reported in previous studies by HRTEM or EDX [41, 42], in which it was found that Si NWs fabricated by the same MACE method could mainly keep their crystal structures and only a thin amorphous layer was observed on the wall surface of the NWs [43, 44]. A thin SiO_2_ layer was found to be formed on the porous nanowire surface, without any other impurities detected on the surface [41].

The photoelectrical measurements on individual Si NWs were carried out with a commercial SPM equipment (Multimode V, Bruker Nano Surfaces), as diagramed in Fig. 1e. In PCAFM, the conductive tip scanned over the sample surface in contact mode with a bias voltage applied between the substrate and electrically grounded tip, and the resulted current was measured. Laser irradiation was introduced into the SPM head through a 400-μm fiber. A 405 nm diode laser with adjustable intensity (DPSS Lasers, MDL-III) was focused onto the substrate, and the laser spot area was about 1 mm^2^ beneath the Pt/Cr-coated tip. For getting the stable current measurements at each laser intensity, we would wait for a few minutes before the measurements to reduce the unstable status caused by laser intensity change as possible. On the other hand, it needed more than ten minutes to complete each current image measurement. As we want to complete the current measurement under different laser intensities before the nanowires were seriously oxidized, laser intensities with relatively large interval (2 W/cm^2^) varied from 0 to 8 W/cm^2^ were chosen. The photoconductive current images as well as I–V curves were measured on individual nanowires under different laser irradiation. By using EFM, both the sample topography and the electrical force-induced phase shift could be recorded by a two-pass mode. In the first pass the topography image was obtained in tapping mode. In the second lifted pass (the tip was lifted high enough to neglect the phase shift induced by van der Waals force), a DC bias was applied between the tip and the sample and the phase shift signal determined by the electrical force gradient was detected. The detailed operation principles could be found in previous studies [45, 46]. Pt/Cr-coated tips (Multi75E-G, Budget Sensors, radius approximately 25 nm) were applied in all electrical measurements and all experiments were performed in a flowing N_2_ ambient. Each sample was pre-dipped in the HF solution (5%) for 30 s to remove the oxide layer on the sample surface, and then the sample was washed in flowing deionized water for at least 5 min so that no HF would remain on the surface, except the Si surface was hydrogen passivated which could protect the Si surface from re-oxidation and maintain semiconductor characteristics for about 60 min [47]. After HF dipping the sample was measured immediately, in order to reduce the influence of the oxide layer on the electrical characterization as possible.

## Results and Discussion

### Photoconductive Property Measurements on Single Si NWs

**Fig. 2:**
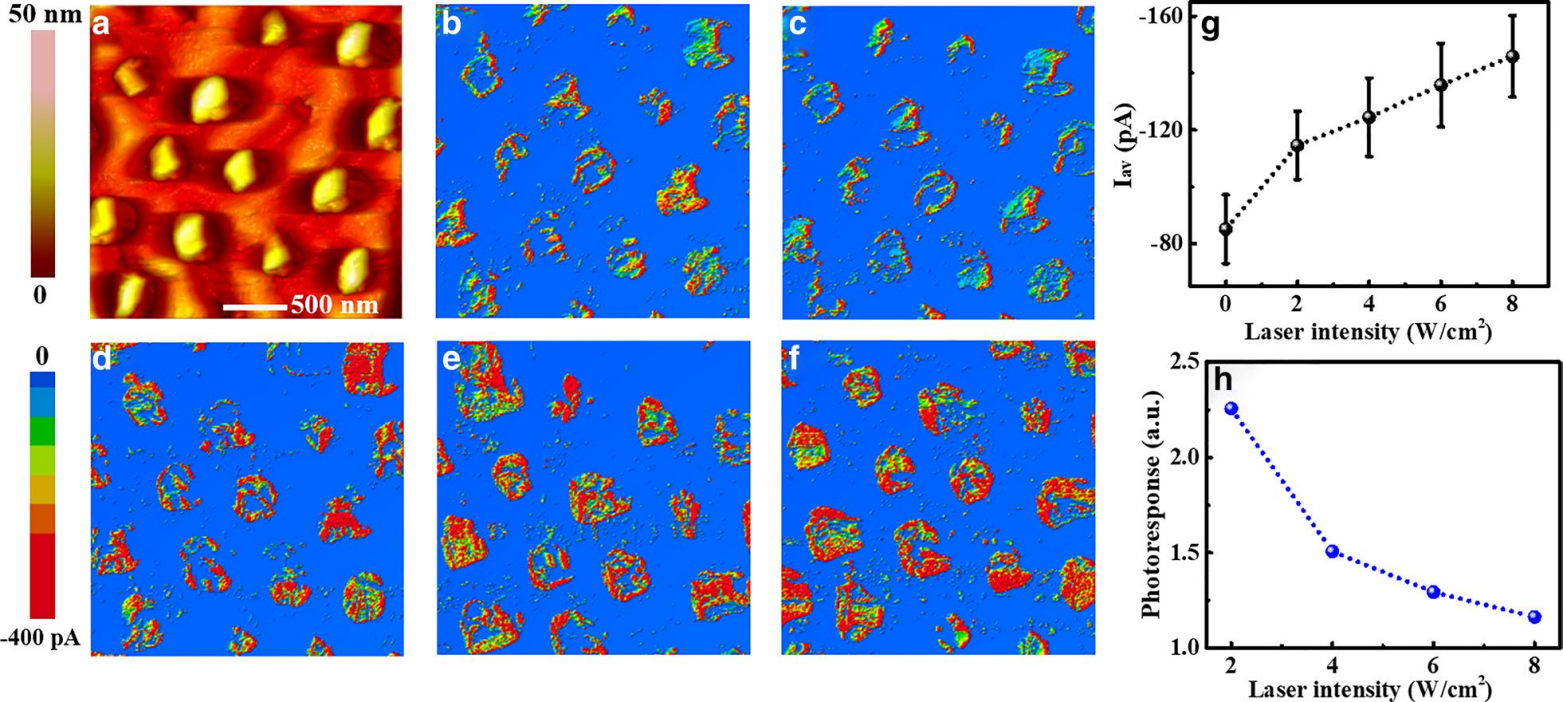
The topography (**a**) and current images of Si NWs with the length of 800 nm and diameter of 190 nm under different laser intensities of **b** 0, **c** 2, **d** 4, **e** 6 and **f** 8 W/cm^2^. **g** presents the averaged current (*I*_av_) over the nanowires as a function of laser intensity. **h** shows the photoresponse as a function of laser intensity

By combining with laser irradiation, the photoconductive properties of Si NWs are investigated by PCAFM as a function of laser intensity. Typical current images obtained on the Si NWs with the diameter of 190 nm and length of 800 nm under different laser irradiation at a sample bias of − 1.5 V are shown in Fig. 2b–f, together with the topography image shown in Fig. 2a. Since the tip was a wedge with a large angle making it unable to reach the bottom, especially the images were obtained in contact mode, the observed nanowires are somewhat distorted and only the current on the top side of nanowires can be measured. Anyways, the current distribution of individual nanowires can be clearly observed from the current images. In the current image without laser irradiation (Fig. 2b), Si NWs exhibit a little better conductance at most of the edges than the center, which was attributed to the larger side contact area between tip and nanowire [40]. Under laser irradiation, the current of Si NWs increases obviously with the laser intensity (Fig. 2c, d), while the conductive area of nanowires increases correspondingly. To get a distinct relation between photocurrent and laser intensity, the average currents of Si NWs are calculated over all the nanowires in the current maps, which are presented in Fig. 2g as a function of laser intensity. The results show that the average current has about two times increase (from 85 to 146 pA) as the laser intensity increases from 0 to 8 W/cm^2^, indicating more carriers are generated under laser irradiation.

In previous studies [32, 48], photoresponse was usually applied to describe the response capability of photodetectors, which was defined as:

1 $$R = \frac{{{{(I_{{\text{L}}} - I_{D} )} \mathord{\left/ {\vphantom {{(I_{{\text{L}}} - I_{D} )} q}} \right. \kern-\nulldelimiterspace} q}}}{{{{P_{{{\text{inc}}}} } \mathord{\left/ {\vphantom {{P_{{{\text{inc}}}} } {h\upsilon }}} \right. \kern-\nulldelimiterspace} {h\upsilon }}}},$$

where *I*_L_ and *I*_D_ are the current with and without laser irradiation, respectively. *P*_inc_ is the product of incident laser power density divided by the effective area of the contact area between the tip and the sample, *q* is the elementary charge and *hν* is the photon energy. In our case the effect contact area is about 2 × 10^–11^ cm^2^ by using the tip radius of 25 nm, and as a result the photoresponse of Si NWs can be calculated to be about 2.3 at the laser intensity of 2 W/cm^2^, indicating that Si NWs have excellent photo enhancement capability. Figure 2h presents the photoresponse as a function of laser intensity, and it can be seen that the photoresponse decreases with the increasing laser intensity but all the values are still larger than 1. Thus, the above results demonstrate that the laser irradiation can greatly enhance the conductance of Si NWs, suggesting its promising application potentials in photodetectors.

**Fig. 3:**
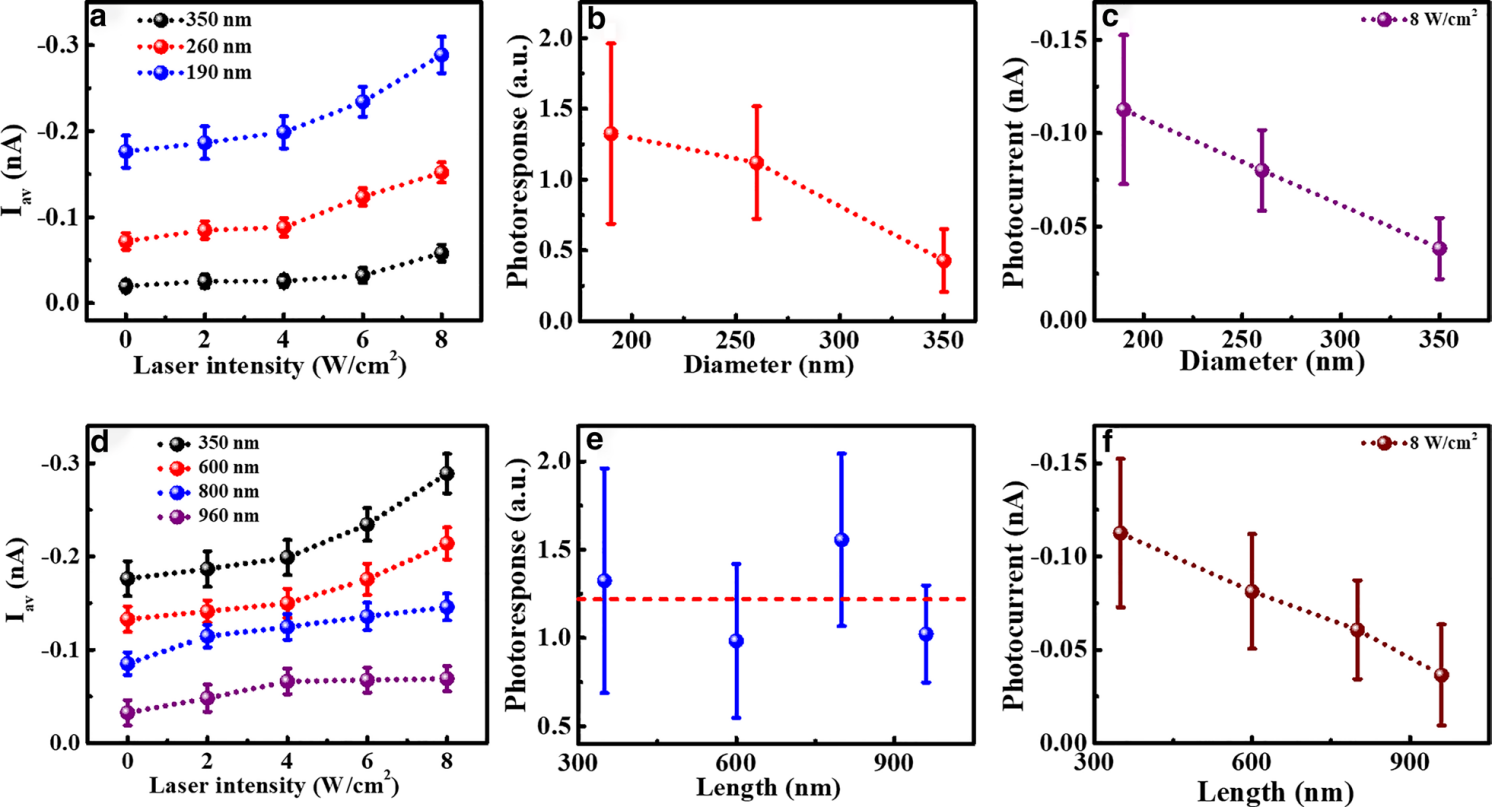
**a** The averaged current (*I*_av_) of Si NWs with different diameters as a function of laser intensity. **b** The averaged photoresponse over the laser intensities as a function of diameters. **c** The dependence of photocurrent on diameter at the laser intensity of 8 W/cm^2^. **d** The *I*_av_ of Si NWs with different lengths as a function of laser intensity. **e** The averaged photoresponse over the laser intensities as a function of length. **f** The dependence of photocurrent on length at the laser intensity of 8 W/cm^2^

To investigate the size dependence of the photoconductive properties, photocurrent measurements were performed on the Si NWs with different diameters and lengths. Typical current images of Si NWs with the same length of 350 nm but different diameters from 190 to 350 nm are shown in Additional file 1: Fig. S1 under 0, 4 and 8 W/cm^2^ laser irradiation at the same sample bias of − 1.5 V. The average currents of Si NWs calculated over all the nanowires in the current images are presented in Fig. 3a as a function of laser intensity. It can be seen that the conductance of Si NWs with all diameters increases obviously with the increased laser intensity. Under the same laser intensity, the absolute current values increase significantly as the diameter decreases from 350 to 190 nm. These results suggest that Si NWs with smaller diameters are more conductive than those with larger ones. The photoresponse averaged over laser intensities is presented in Fig. 3b for different diameters. It can be seen that the photoresponse decreases with the increased diameter, which means that Si NWs with smaller diameters have better photoresponse capability. On the other hand, the photocurrent (*I*_L_ − *I*_D_) at the laser intensity of 8 W/cm^2^ for different diameters is shown in Fig. 3c. It clearly shows that the photocurrent decreases as the diameter increases, indicating that Si NWs with smaller diameters have better photoconductance.

Similar measurements are carried out on Si NWs with same diameter but different lengths. The results of nanowires with the diameter of 190 nm and lengths from 350 to 960 nm are shown in Additional file 1: Fig. S2. The average currents of nanowires with different lengths are presented in Fig. 3d. With the increased laser intensity, all of the nanowires exhibit an obvious increase in conductance, and the shorter Si NWs have the larger conductance across the laser intensity range up to 8 W/cm^2^. The photoresponse and photocurrent as a function of nanowires’ length at the laser intensity of 8 W/cm^2^ are presented in Fig. 3e, f, respectively. It can be seen that with the increase of length from 350 to 960 nm, the photoresponse does not show an obvious length dependence, while the photocurrent largely decreases with the increase of length.

### I–V Curves Analysis and Size-Dependent Schottky Barrier Height

**Fig. 4:**
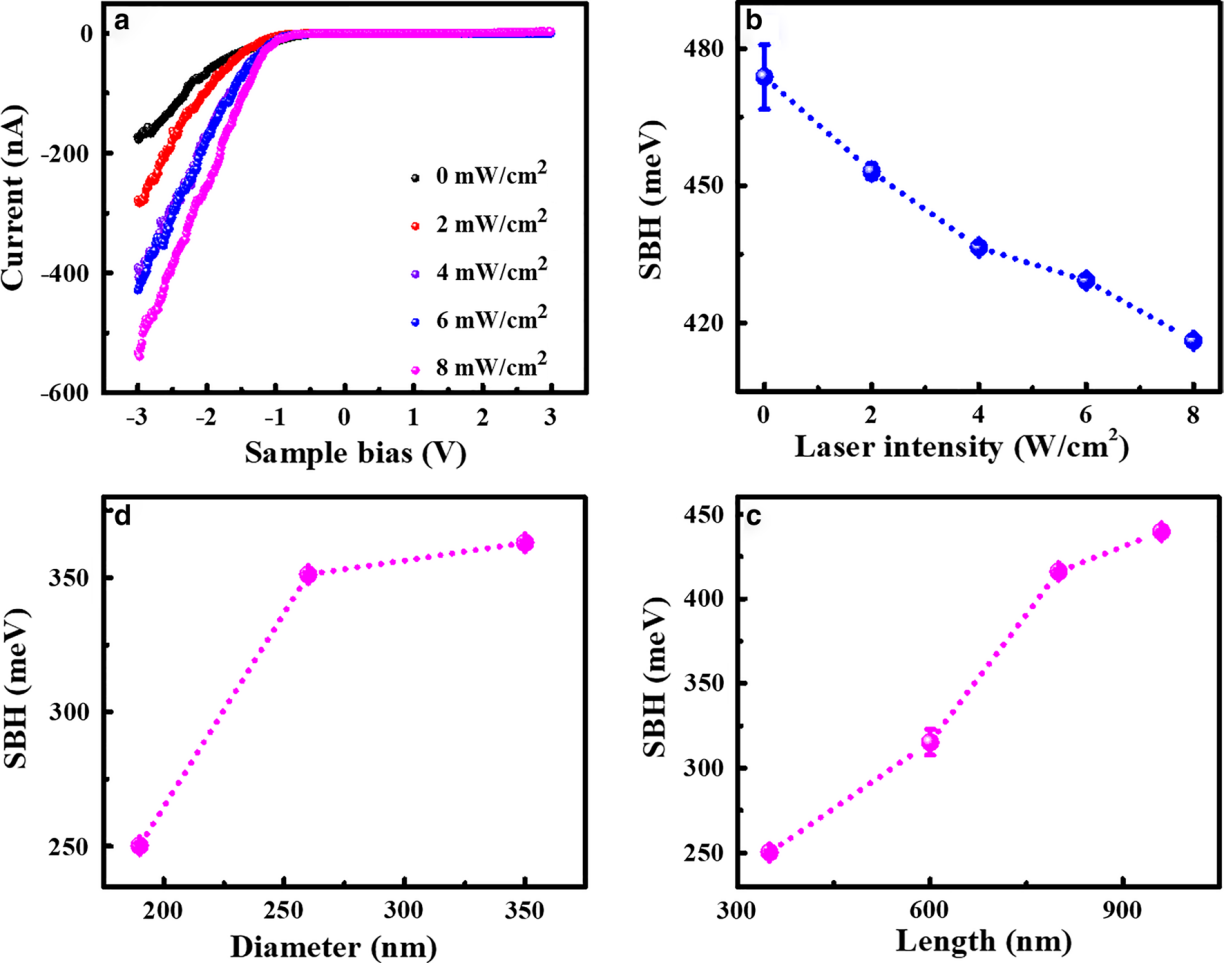
**a** Typical I–V curves of Si NWs with 190 nm in diameter and 800 nm in length under different laser irradiation. **b** SBH values obtained from the fitting of I–V curves in **a**. The diameter- and length-dependent SBH values under 8 W/cm^2^ laser irradiation are plotted in **c**, **d**, respectively

As reported in our previous work [40], in CAFM measurements on Si NWs, tip-nanowire contact resistance should be emphatically considered, in which the Schottky barrier plays an important role. To investigate the role of Schottky barrier in photoconductance and the effect of laser irradiation on barrier height, current–voltage (I–V) curves are recorded on individual Si NWs. Typical I–V curves on the Si NWs with the diameter of 190 nm and length of 800 nm under different laser irradiation are presented in Fig. 4a. All the I–V curves exhibit typical I–V characteristic of metal and n-type semiconductor contact, indicating the effect of oxygen layer on conductance is not serious and thus ignored in the following discussion. It can be observed that, as the laser intensity increases, the current of the Si NWs increases obviously. The enhancement can reach about 3 times when the laser intensity increases from 0 to 8 W/cm^2^ under the bias of -1.5 V, which is well consistent with the results obtained from current images. To get a quantitative analysis, a well-known thermionic emission model for a metal–semiconductor contact is adopted [13, 49]. In this model, the I–V characteristics of a Schottky contact to n-type semiconductor in the presence of series resistance can be approximated as [13]:

2 $$I = I_{{\text{S}}} \left[ {\exp \left( {\frac{{q(V - IR_{{\text{S}}} )}}{{{\text{n}}kT}}} \right) - {1}} \right],$$

where *n* is the ideal factor and *R*_S_ is the series resistance. *I*_S_ is the saturation current, which can be expressed by:

3 $$I_{S} = AA^{*} T^{2} \exp \left( { - \frac{{\user2{\varphi }_{{\text{B}}} }}{kT}} \right),$$

where *A* is the contact area, $A^{*}$ is Richardson’s constant, and ***φ***_B_ is the Schottky barrier height (SBH) between the metal tip and Si nanowire. Thus, SBH can be obtained with the formula:

4 $$\user2{\varphi }_{{\text{B}}} = kT\ln \left( {\frac{{AA^{*} T^{2} }}{{I_{{\text{S}}} }}} \right),$$

The I–V curves in Fig. 4a can be well fitted by Eq. (2). To get the SBH values from the saturation current, the effective Richardson constant $A^{*}$ is assumed to be approximately equal to that of bulk silicon, i.e. 112 A cm^−2^ K^−2^ for n-type silicon. The contact area is assumed to be 2 × 10^–11^ cm^2^ by taking the Cr/Pt coated tip radius as 25 nm. The SBH values are obtained to be about 474, 453, 437, 429 and 416 meV for different laser intensities of 0, 2, 4, 6 and 8 W/cm^2^, respectively, as plotted in Fig. 4b. It demonstrates that SBH decreases significantly with the laser intensity, which may be the main contributor to the photoenhanced conductance. At the meantime, the dependence of SBH on the nanowires’ diameter and length at the same laser intensity is given in Fig. 4c, d, respectively. The results indicate that Si NWs with smaller diameters and shorter lengths have smaller SBH values, resulting in better photoconductance obtained on such nanowires. The diameter and length dependence of SBH under different laser irradiation are shown in Additional file 1: Fig. S3, which further supports the above conclusion. Obviously, all of the measured SBH values for Si NWs with different diameters and lengths are smaller than that of the bulk Si (~ 600 meV) [40] and further decrease with the increased laser intensity, indicating that Si NWs can achieve promising photoconductive properties for potential applications.

Therefore, from the above results, it can be concluded that the photoconductive properties of Si NWs are strongly dependent on their diameters and lengths, i.e. Si NWs with smaller diameters and shorter lengths exhibit better photoconductance, which should be attributed the size-dependent SBH as revealed by I–V curve fitting. The exact mechanism about the size dependence of SBH is not clear yet. It may be related to the interface states and/or disordered structure in the rough outer layer. According to the previous studies [50–52], charged interface states could effectively reduce SBH. For example, in reference [50], Yoon et al. supposed that the interface state-induced carrier transfer would form two opposite charged layers with negatively charged surface states and the equal number of positive charges, which could generate an electric field in contrary to the built-in electric field, resulting in an effective lowering of SBH that was strongly dependent on nanowires’ diameter. By using finite element modeling and treated the nanowire as a cylindrical coaxial capacitor, they found that the magnitude of barrier lowering would increases as the nanowire diameter was decreased. In our case, due to the rough surface of MACE fabricated nanowires, when contacting with the metal tip, a large density of interface states would be generated which can also effectively lower the barrier height by adopting the above viewpoint. The surface state density increases with decreased nanowire diameter, smaller SBH can be achieved on the nanowires with smaller diameters. Thus, Si NWs with smaller diameters exhibit larger conductance. Because SBH decreases with laser intensity for all diameters, Si NWs with smaller diameters also exhibit larger photoconductance.

The reason why the values of SBH is length dependent, however, could not be interpreted with this viewpoint. Longer nanowires require more MACE time to fabricate, resulting in more surface disorder or roughness. Different changes in the surface microstructures may introduce different changes of SBH values, which need further investigations to work it out. Anyway, whatever the origin of size dependence of photoconductive properties, the size-dependent SBH lowering could result in higher conductance or photoconductance, which should be beneficial for practical applications.

### Photogenerated Trapped Charges and Barrier Height Modification

**Fig. 5:**
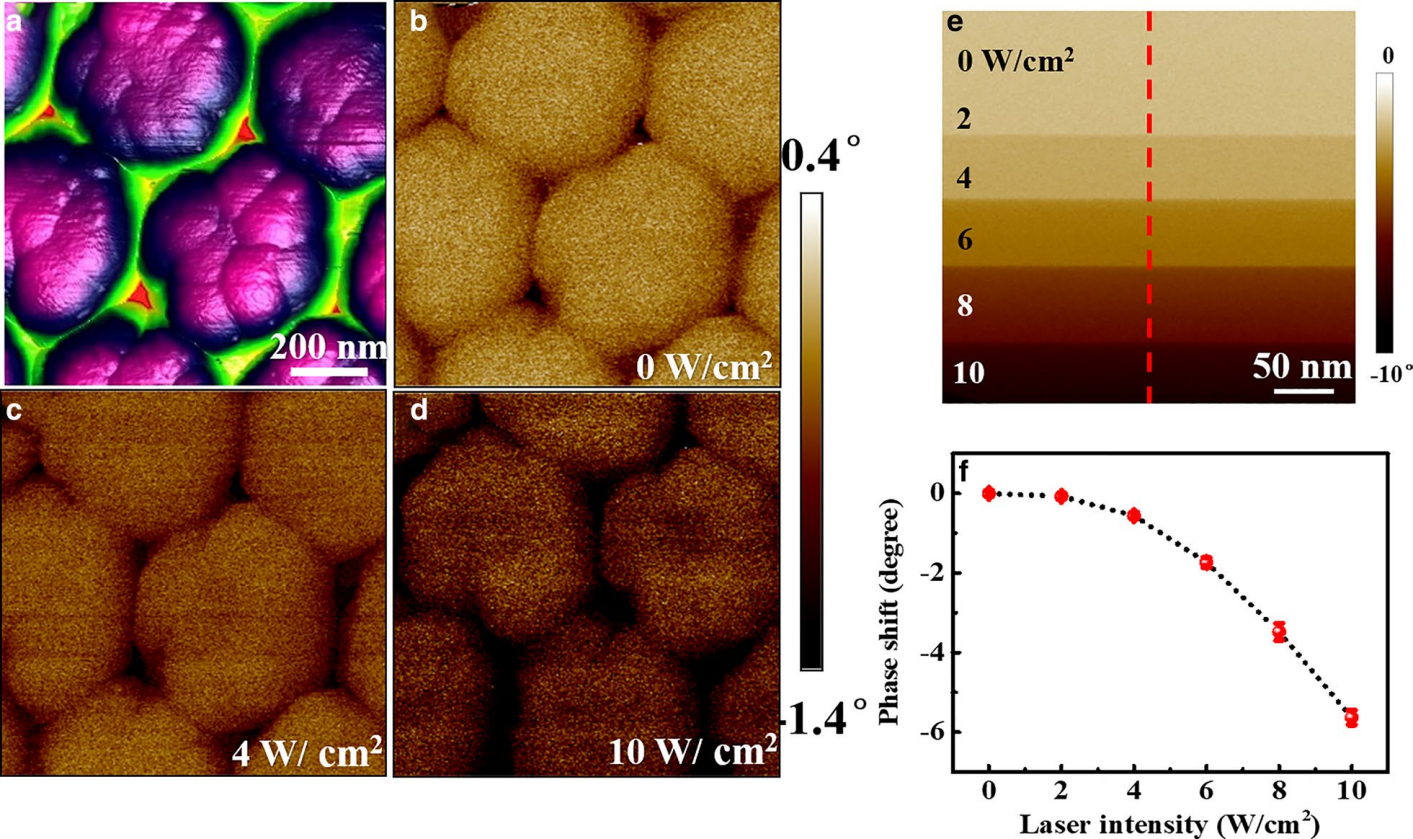
The topography image of Si NWs (**a**), the phase shift images obtained at different laser intensities of 0 (**b**), 4 (**c**) and 10 W/cm^2^ (**d**), respectively. **e** The phase shift image acquired in line scan mode on the top center of nanowire. The averaged phase shift over the scan line along the marked red curve in **e** is plotted in **f**

To further verify the SBH results of Si NWs obtained by PCAFM, EFM images were measured on Si NWs under different laser irradiation, as shown in Fig. 5a–d. It can be seen that the electrostatic force induced phase shift (Δ*Φ*) increases obviously with the laser intensity. The phase shift image acquired in line scan mode on the top center of the nanowire is presented in Fig. 5e, and the averaged phase shift over the scan line along the marked curve is drawn in Fig. 5f. Both of them clearly exhibit the increase of Δ*Φ* with laser intensity.

**Fig. 6:**
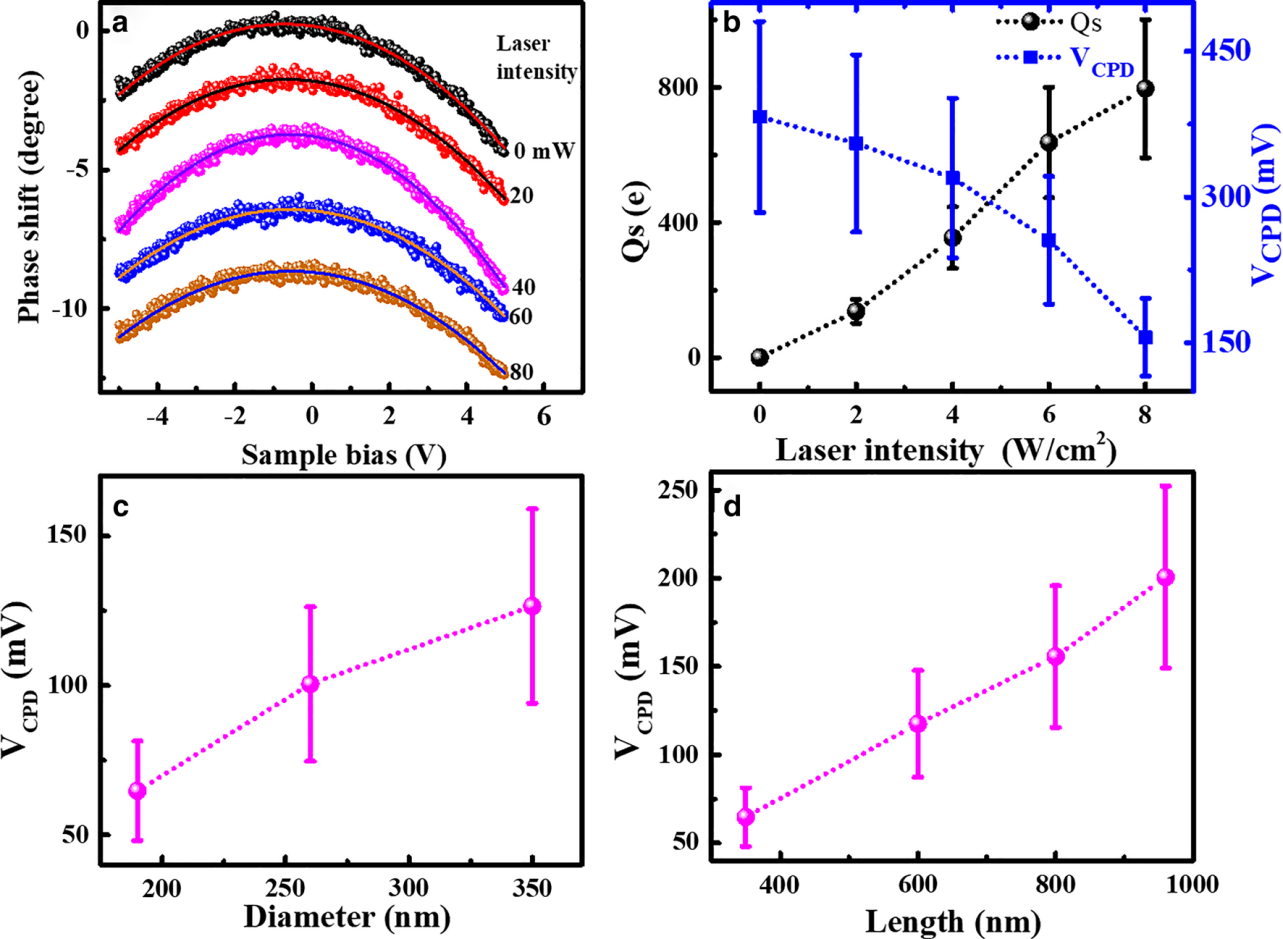
**a** ∆*Φ* ~ *V*_EFM_ curves measured by EFM on individual Si NWs with the diameter of 190 nm and length of 800 nm under different laser irradiation. **b** The results of *Qs* and *V*_CPD_ obtained by fitting the curves in **a** as a function of laser intensity. The diameter and length dependence of *V*_CPD_ at the laser intensity of 8 W/cm^2^ is presented in **c**, **d**, respectively

To get more definite information from EFM measurements, Δ*Φ* was measured as a function of applied voltage (*V*_EFM_) under different laser irradiation on a certain single nanowire. A set of Δ*Φ* ~ *V*_EFM_ curves measured on the Si nanowire with the diameter of 190 nm and the length of 800 nm are presented in Fig. 6a as the scattered dots. It can be seen that, with the increase of laser intensity, the Δ*Φ* ~ *V*_EFM_ curves shift downward. It indicates more carriers are generated and trapped in nanowires [45]. For quantitative analysis, the tip-sample system is simply treated as a plane capacitor, and the capacitive electrostatic force gradient would cause a phase shift when applying a bias between the tip and sample. With charges trapped in the nanostructures by laser irradiation, additional phase shift induced by the Coulombic force would be generated [53]. The phase shift detected by EFM can be described as [54, 55]:

5 $$\Delta \Phi = - \frac{Q}{k}\frac{\partial F}{{\partial z}} = - \frac{Q}{k}\left[ {\frac{1}{2}\frac{{\partial^{2} C}}{{\partial z^{2} }}(V_{{{\text{EFM}}}} - V_{{{\text{CPD}}}} )^{2 \, } + \frac{{Q_{{\text{s}}} }}{{2\pi \varepsilon_{0} z^{2} }}\left( {\frac{C}{z} - \frac{1}{2}\frac{\partial C}{{\partial z}}} \right)(V_{{{\text{EFM}}}} - V_{{{\text{CPD}}}} ) + \frac{{Q_{{\text{s}}}^{2} }}{{2\pi \varepsilon_{0} z^{3} }}} \right],$$

where *C*, *V*_EFM_ and *V*_CPD_ are the capacitance, applied DC voltage and contact potential difference between the tip and sample, respectively. *Q*_s_ is the quantity of charges trapped in the nanowire, *Q* is the quality factor and *k* is the spring constant of the probe, and *z* is the distance between the trapped charges in nanowire.

Δ*Φ* ~ *V*_EFM_ curves in Fig. 6a can be well fitted by using Eq. (5), shown as the solid lines. From the fitting parameters, *V*_CPD_ and *Q*_s_ can be obtained using *Q* = 186 and *k* = 2.8 N/m for Pt/Ir-coated tip [56, 57] and approximating *z* as the lift height, which are plotted in Fig. 6b as a function of laser intensity. It can be seen that, with the increase of laser intensity, *V*_CPD_ decreases while the trapped charges *Q*_s_ increase. As reported in literature [46], the change of *V*_CPD_ under laser irradiation was related to the variation in trapped carrier density. Thus the decrease of *V*_CPD_ with laser irradiation in our experiments can also be attributed to the increase of trapped charge density.

From the energy diagram given in Additional file 1: Fig. S4, the value of SBH roughly equals to *qV*_CPD_ plus *E*_n_ (= *E*_C_ − *E*_F_) [40]. As *E*_n_ is a constant for all Si NWs made from the same material, the size dependence of *V*_CPD_ well represents that of SBH. The results of *V*_CPD_ obtained on Si NWs with different diameters and lengths are presented in Additional file 1: Fig. S5 as a function of laser intensity. All of the measured *V*_CPD_ for Si NWs with different diameters and lengths decrease with the increased laser intensity. The dependence of *V*_CPD_ on nanowires’ diameter and length at the same laser intensity of 8 W/cm^2^ is shown in Fig. 6c, d, respectively. It can be seen that the *V*_CPD_ increases obviously with the increased diameter and increased length, in a good agreement with the size dependence of SBH. Therefore, from the EFM results, it can be suggested the laser irradiation can generate carriers trapped in nanowires, which can induce the lowering of barrier height leading to the enhancement of conductance (Fig. 4).

## Conclusion

In summary, by a simple and low-cost method without involving any intricated procedures, Si NWs arrays with controllable diameters and lengths are prepared. The photoconductive properties are directly measured on individual Si NWs without complex nanofabrication procedure by the means of PCAFM. The size-dependent conductance of Si NWs is obtained on individual nanowires with different diameters and lengths. The results demonstrate that the photocurrent measured on individual Si NWs increases greatly with the laser intensity, and the increasing magnitude is obviously related to the nanowires’ sizes. Si NWs with smaller diameters and shorter lengths exhibit larger photoconductance. On the other hand, the measurements performed by EFM combined with laser irradiation provided the information of photogenerated charges and contact barrier height, which can be applied to explain the photoconductive properties of Si NWs as well as their size-dependence. Therefore, in this study, the photoelectrical properties are investigated on individual nanowires by PCAFM and EFM, which should be important for both basic understanding and potential applications of nanostructures in optoelectronics and photovoltaics.

## Supplementary Information

**Additional file 1.** **Fig. S1**. The current images of Si NWs with the same length but different diameters. **Fig. S2.** The current images of Si NWs with the same diameter but different lengths under different laser intensities. **Fig. S3.** The Schottky barrier heights obtained from the fitting results as a function of laser intensity for Si NWs with different diameters and different lengths respectively. **Fig. S4.** Energy band diagram of the contact interface between the metallic tip and n-type Si nanowire. **Fig. S5.** Contact potential difference (VCPD) obtained from the fitting results as a function of laser intensity on Si NWs with different diameters and different lengths.

## Data Availability

The datasets used for supporting the conclusion are included in the article and the supporting file.

## Abbreviations

Si NWs: Si nanowires
CAFM: Conductive atomic force microscopy
PCAFM: Photoconductive conductive atomic force microscopy
EFM: Electrostatic force microscopy
PV: Photovoltaic
NSL: Nanosphere lithography
MACE: Metal-assisted chemical etching
SPM: Scanning probe microscopy
PS: Polystyrene spheres
RIE: Reactive ion etching
SEM: Scanning electron microscopy
EDX: Energy dispersive X-ray spectroscopy
SBH: Schottky barrier height
CPD: Contact potential difference

## References

[1] Lin D, Wu Z, Li S, Zhao W, Ma C, Wang J, Jiang Z, Zhong Z, Zheng Y, Yang X (2017). Large-area Au-nanoparticle-functionalized Si nanorod arrays for spatially uniform surface-enhanced Raman spectroscopy. ACS Nano.

[2] Lard M, Linke H, Prinz CN (2019). Biosensing using arrays of vertical semiconductor nanowires: mechanosensing and biomarker detection. Nanotechnology.

[3] Fukata N, Subramani T, Jevasuwan W, Dutta M, Bando Y (2017). Functionalization of silicon nanostructures for energy-related applications. Small.

[4] Yang P, Yan R, Fardy M (2010). Semiconductor nanowire: what's next?. Nano Lett.

[5] Su X, Wu Q, Li J, Xiao X, Lott A, Lu W, Sheldon BW, Wu J (2014). Silicon-based nanomaterials for lithium-ion batteries: a review. Adv Energy Mater.

[6] Long Y-Z, Yu M, Sun B, Gu C-Z, Fan Z (2012). Recent advances in large-scale assembly of semiconducting inorganic nanowires and nanofibers for electronics, sensors and photovoltaics. Chem Soc Rev.

[7] Tsakalakos L (2008). Nanostructures for photovoltaics. Mater Sci Eng R Rep.

[8] Soci C, Zhang A, Bao X-Y, Kim H, Lo Y, Wang D (2010). Nanowire photodetectors. J Nanosci Nanotechnol.

[9] Peng K-Q, Wang X, Li L, Hu Y, Lee S-T (2013). Silicon nanowires for advanced energy conversion and storage. Nano Today.

[10] Wang Y, Wang T, Da P, Xu M, Wu H, Zheng G (2013). Silicon nanowires for biosensing, energy storage, and conversion. Adv Mater.

[11] LaPierre RR, Chia ACE, Gibson SJ, Haapamaki CM, Boulanger J, Yee R, Kuyanov P, Zhang J, Tajik N, Jewell N, Rahman KMA (2013). III–V nanowire photovoltaics: review of design for high efficiency. Phys Status Solidi-R.

[12] Yuan G, Zhao H, Liu X, Hasanali ZS, Zou Y, Levine A, Wang D (2009). Synthesis and photoelectrochemical study of vertically aligned silicon nanowire arrays. Angew Chem Int Ed Engl.

[13] Yang Z, Du K, Wang H, Lu F, Pang Y, Wang J, Gan X, Zhang W, Mei T, Chua SJ (2019). Near-infrared photodetection with plasmon-induced hot electrons using silicon nanopillar array structure. Nanotechnology.

[14] Giridharagopa R, Shao G, Groves C, Ginger DS (2010). New SPM techniques for analyzing OPV materials. Mater Today.

[15] Hui F, Lanza M (2019). Scanning probe microscopy for advanced nanoelectronics. Nat Electron.

[16] Liu J, Goswami A, Jiang K, Khan F, Kim S, McGee R, Li Z, Hu Z, Lee J, Thundat T (2018). Direct-current triboelectricity generation by a sliding Schottky nanocontact on MoS2 multilayers. Nat Nanotechnol.

[17] Yang T, Hertenberger S, Morkötter S, Abstreiter G, Koblmüller G (2012). Size, composition, and doping effects on In(Ga)As nanowire/Si tunnel diodes probed by conductive atomic force microscopy. Appl Phys Lett.

[18] Tang C, Jiang C, Bi S, Song J (2016). Photoelectric property modulation by nanoconfinement in the longitude direction of short semiconducting nanorods. ACS Appl Mater Interfaces.

[19] Alvarez J, Ngo I, Gueunier-Farret ME, Kleider JP, Yu L, Cabarrocas PR, Perraud S, Rouviere E, Celle C, Mouchet C, Simonato JP (2011). Conductive-probe atomic force microscopy characterization of silicon nanowire. Nanoscale Res Lett.

[20] Xia H, Lu Z-Y, Li T-X, Parkinson P, Liao Z-M, Liu F-H, Lu W, Hu W-D, Chen P-P, Xu H-Y, Zou J, Jagadish C (2012). Distinct photocurrent response of individual GaAs nanowires induced by n-type doping. ACS Nano.

[21] O’Dea JR, Brown LM, Hoepker N, Marohn JA, Sadewasser S (2012). Scanning probe microscopy of solar cells: from inorganic thin films to organic photovoltaics. MRS Bull.

[22] Dang X-D, Tamayo AB, Seo J, Hoven CV, Walker B, Nguyen T-Q (2010). Nanostructure and optoelectronic characterization of small molecule bulk heterojunction solar cells by photoconductive atomic force microscopy. Adv Funct Mater.

[23] Guide M, Dang XD, Nguyen TQ (2011). Nanoscale characterization of tetrabenzoporphyrin and fullerene-based solar cells by photoconductive atomic force microscopy. Adv Mater.

[24] Coffey DC, Reid OG, Rodovsky DB, Bartholomew GP, Ginger DS (2007). Mapping local photocurrents in polymerfullerene solar cells with photoconductive atomic force microscopy. Nano Lett.

[25] Hamadani BH, Jung S, Haney PM, Richter LJ, Zhitenev NB (2010). Origin of nanoscale variations in photoresponse of an organic solar cell. Nano Lett.

[26] Kamkar DA, Wang M, Wudl F, Nguyen T-Q (2012). Single nanowire OPV properties of a fullerene-capped P3HT dyad investigated using conductive and photoconductive AFM. ACS Nano.

[27] Heo J-H (2012). Characterization of photoinduced current in poly-Si solar cell by employing photoconductive atomic force microscopy (PC-AFM). Trans Electr Electron Mater.

[28] Heo J (2013). Characterization of wavelength effect on photovoltaic property of poly-Si solar cell using photoconductive atomic force microscopy (PC-AFM). Trans Electr Electron Mater.

[29] Zhao Z, Chen X, Wu H, Wu X, Cao G (2016). Probing the photovoltage and photocurrent in perovskite solar cells with nanoscale resolution. Adv Funct Mater.

[30] Ledinský M, Fejfar A, Vetushka A, Stuchlík J, Rezek B, Kočka J (2011). Local photoconductivity of microcrystalline silicon thin films measured by conductive atomic force microscopy. Phys Status Solidi-R.

[31] Li Z, Li H, Jiang K, Ding D, Li J, Ma C, Jiang S, Wang Y, Anthopoulos TD, Shi Y (2019). Self-powered perovskite/CdS heterostructure photodetectors. ACS Appl Mater Interfaces.

[32] Son Y, Wang QH, Paulson JA, Shih C-J, Rajan AG, Tvrdy K, Kim S, Alfeeli B, Braatz RD, Strano MS (2015). Layer number dependence of MoS2 photoconductivity using photocurrent spectral atomic force microscopic imaging. ACS Nano.

[33] Fan Z, Dutta D, Chien C-J, Chen H-Y, Brown EC, Chang P-C, Lu JG (2006). Electrical and photoconductive properties of vertical ZnO nanowires in high density arrays. Appl Phys Lett.

[34] Sanford NA, Robins LH, Blanchard PT, Soria K, Klein B, Eller BS, Bertness KA, Schlager JB, Sanders AW (2013). Studies of photoconductivity and field effect transistor behavior in examining drift mobility, surface depletion, and transient effects in Si-doped GaN nanowires in vacuum and air. J Appl Phys.

[35] Kim CJ, Lee HS, Cho YJ, Kang K, Jo MH (2010). Diameter-dependent internal gain in ohmic Ge nanowire photodetectors. Nano Lett.

[36] Wu S, Yu H, Lu N, Quan X, Chen S (2017). Fabrication of the hierarchical structure photocathode by structuring the surface nanopores on Si nanowires standing on p-Si wafer for the effective photoelectrochemical reduction of Cr(VI) in the aqueous solution. Sep Purif Technol.

[37] Wu SL, Wen L, Cheng GA, Zheng RT, Wu XL (2013). Surface morphology-dependent photoelectrochemical properties of one-dimensional Si nanostructure arrays prepared by chemical etching. ACS Appl Mater Interfaces.

[38] Dong F, Chu V, Conde JP (2011). Submicron thin-film amorphous silicon photoconductive light sensors. Senser Actuat A-Phys.

[39] Calarco R, Marso M (2005). Size-dependent photoconductivity in MBE-grown GaN-nanowires. Nano Lett.

[40] Hu XF, Li SJ, Wang J, Jiang ZM, Yang XJ (2020). Investigating size-dependent conductive properties on individual Si nanowires. Nanoscale Res Lett.

[41] Weidemann S, Kockert M, Wallacher D, Ramsteiner M, Mogilatenko A, Rademann K, Fischer SF (2015). Controlled pore formation on mesoporous single crystalline silicon nanowires: threshold and mechanisms. J Nanomater.

[42] Peng K, Lu A, Zhang R, Lee S-T (2008). Motility of metal nanoparticles in silicon and induced anisotropic silicon etching. Adv Funct Mater.

[43] Zhang M-L, Peng K-Q, Fan X, Jie J-S, Zhang R-Q, Lee S-T, Wong N-B (2008). Preparation of large-area uniform silicon nanowires arrays through metal-assisted chemical etching. J Phys Chem C.

[44] Chen H, Zou R, Chen H, Wang N, Sun Y, Tian Q, Wu J, Chen Z, Hu J (2011). Lightly doped single crystalline porous Si nanowires with improved optical and electrical properties. J Mater Chem.

[45] Altvater MA, Wu S, Zhang Z, Zhu T, Li G, Watanabe K, Taniguchi T, Andrei EY (2019). Electrostatic imaging of encapsulated grapheme. 2D Mater.

[46] Wu S, Wu Z, Lin D, Zhong Z, Jiang Z, Yang X (2014). Photogenerated charges and surface potential variations investigated on single Si nanorods by electrostatic force microscopy combined with laser irradiation. Nanoscale Res Lett.

[47] Wu R, Li FH, Jiang ZM, Yang XJ (2006). Effects of a native oxide layer on the conductive atomic force microscopy measurements of self-assembled Ge quantum dots. Nanotechnology.

[48] Son Y, Li M-Y, Cheng C-C, Wei K-H, Liu P, Wang QH, Li L-J, Strano MS (2016). Observation of switchable photoresponse of a monolayer WSe2-MoS2 lateral heterostructure via photocurrent spectral atomic force microscopic imaging. Nano Lett.

[49] Park JY, Lee H, Renzas JR, Zhang Y, Somorjai GA (2008). Probing hot electron flow generated on Pt nanoparticles with Au TiO2 Schottky diodes during catalytic CO oxidation. Nano Lett.

[50] Yoon K, Hyun JK, Connell JG, Amit I, Rosenwaks Y, Lauhon LJ (2013). Barrier height measurement of metal contacts to Si nanowires using internal photoemission of hot carriers. Nano Lett.

[51] Koren E, Berkovitch N, Azriel O, Boag A, Rosenwaks Y, Hemesath ER, Lauhon LJ (2011). Direct measurement of nanowire Schottky junction depletion region. Appl Phys Lett.

[52] Piscator J, Engström O (2008). Schottky barriers on silicon nanowires influenced by charge configuration. Appl Phys Lett.

[53] Dokukin M, Olac-Vaw R, Guz N, Mitin V, Sokolov I (2009). Addressable photocharging of single quantum dots assisted with atomic force microscopy probe. Appl Phys Lett.

[54] Lei CH, Das A, Elliott M, Macdonald JE (2004). Quantitative electrostatic force microscopy-phase measurements. Nanotechnology.

[55] Jespersen TS, Nygård J (2005). Charge trapping in carbon nanotube loops demonstrated by electrostatic force microscopy. Nano Lett.

[56] Yalcin SE, Labastide JA, Sowle DL, Barnes MD (2011). Spectral properties of multiply charged semiconductor quantum dots. Nano Lett.

[57] Yalcin SE, Yang B, Labastide JA, Barnes MD (2012). Electrostatic force microscopy and spectral studies of electron attachment to single quantum dots on indium tin oxide substrates. J Phys Chem C.

