# Supplementary material for: Photoelectrical Properties Investigated on Individual Si Nanowires and Their Size Dependence
Source: Nanoscale Res Lett. 2021 Jan 28;16:18. doi: 10.1186/s11671-021-03487-1 (PMC7843832; doi:10.1186/s11671-021-03487-1)
Supplement: Supplementary file 1 — Additional file 1. Fig. S1. The current images of Si NWs with the same length but different diameters. Fig. S2. The current images of Si NWs with the same diameter but different lengths under different laser intensities. Fig. S3. The Schottky barrier heights obtained from the fitting results as a function of laser intensity for Si NWs with different diameters and different lengths respectively. Fig. S4. Energy band diagram of the contact interface between the metallic tip and n-type Si nanowire. Fig. S5. Contact potential difference (VCPD) obtained from the fitting results as a function of laser intensity on Si NWs with different diameters and different lengths. [file 11671_2021_3487_MOESM1_ESM.pdf]

Photoelectrical properties investigated on individual Si nanowires and their size dependence

Xiaofeng Hu[1,2], Shujie Li[1,2], Zuimin Jiang[1] and Xinju Yang[1*]

[1]*State Key Laboratory of Surface Physics, Fudan University, Shanghai 200433, China*

[2]*Kunming Institute of Physics, Kunming 650223, China*

[*] Corresponding author. E-mail address: xjyang@fudan.edu.cn.

1. Current images of Si NWs with different diameters under different laser irradiation

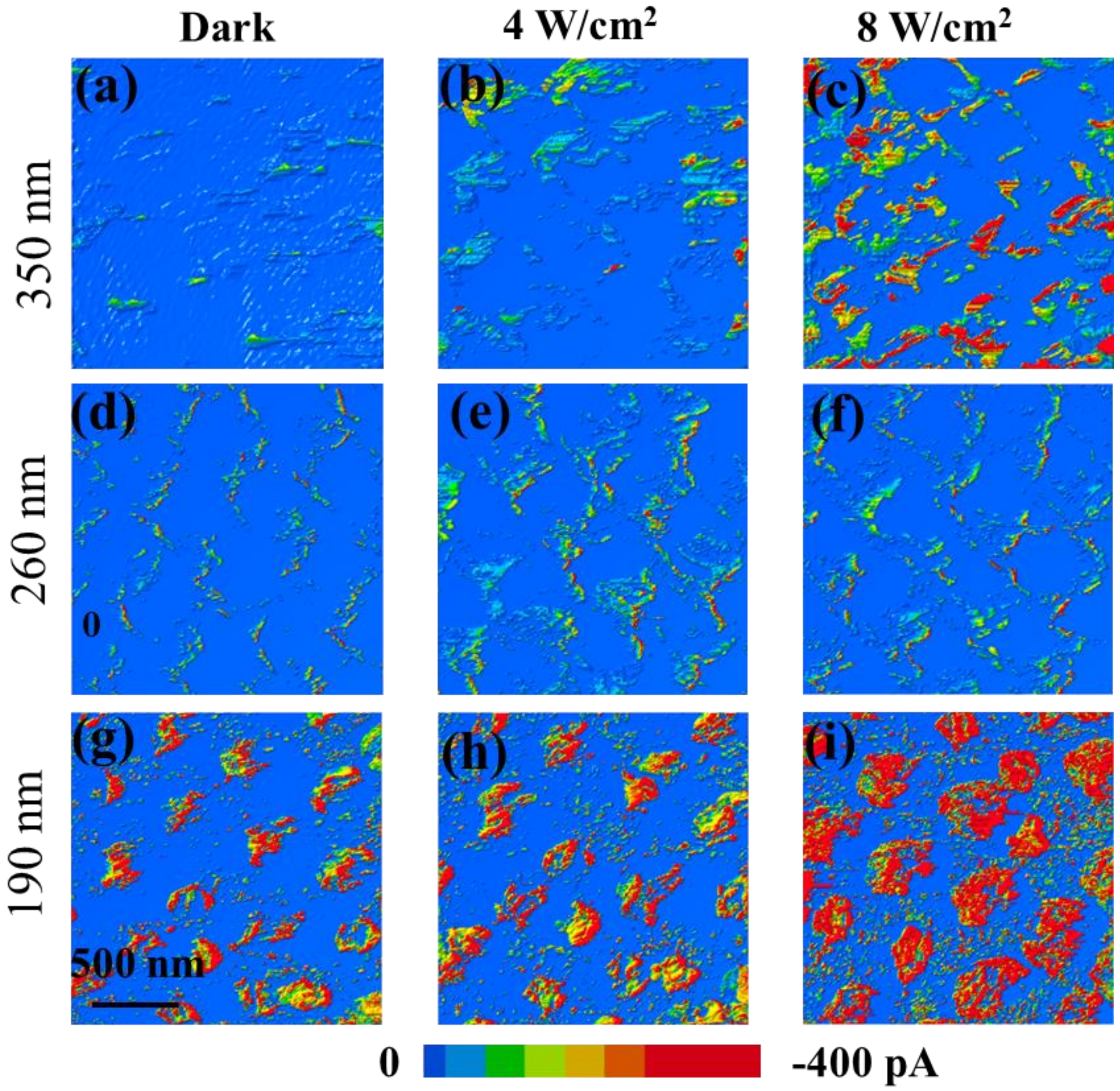

Fig. S1 The current images of Si NWs with the same length of 350 nm but different diameters: (a)-(c) 350 nm; (d)-(f) 260 nm; and (g)-(i) 190 nm under the laser intensities of 0, 4 and 8 W/cm^2.

2. Current images of Si NWs with different lengths under different laser irradiation

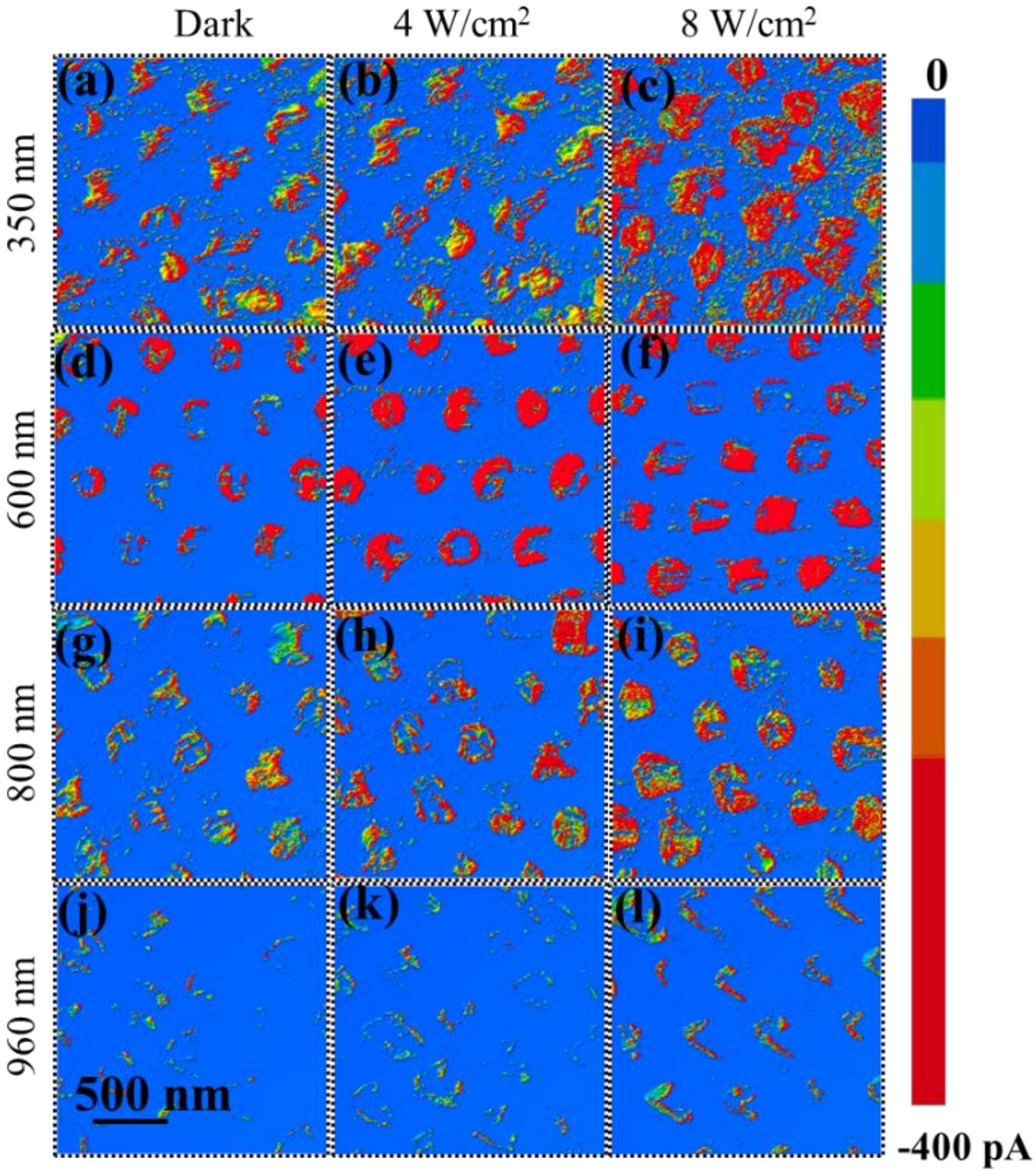

Fig. S2 The current images of Si NWs under the laser intensity of 0, 4 and 8 W/cm^2 with the same diameter of 190 nm but different lengths: 350 nm: (a)-(c); 600 nm: (d)-(f); 800 nm:(g)-(i); 960 nm: (j)-(l).

3. Schottky barrier heights of Si NWs with different diameters and lengths

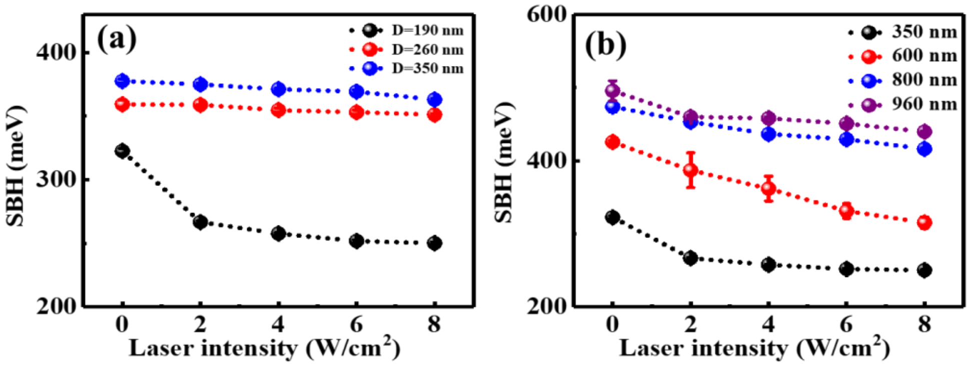

Fig. S3 The Schottky barrier heights obtained from the fitting results as a function of laser intensity for Si NWs with different diameters (a) and different lengths (b), respectively.

4. Schematic energy band diagram of the tip-sample contact

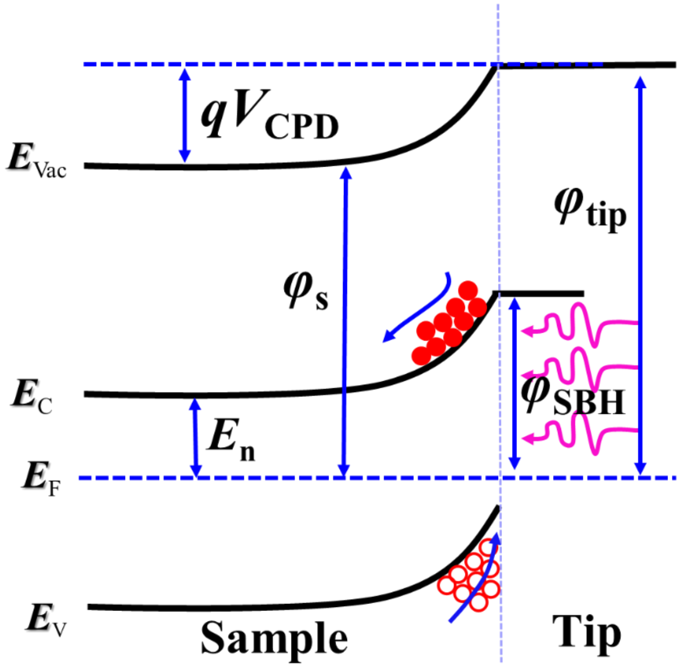

Fig. S4 Energy band diagram of the contact interface between the metallic tip and n-type Si nanowire. V_{CPD} is the contact potential difference and the value of SBH roughly equals to the sum of qV_{CPD} and E_n.

5. Contact potential difference results obtained on different series of Si NWs.

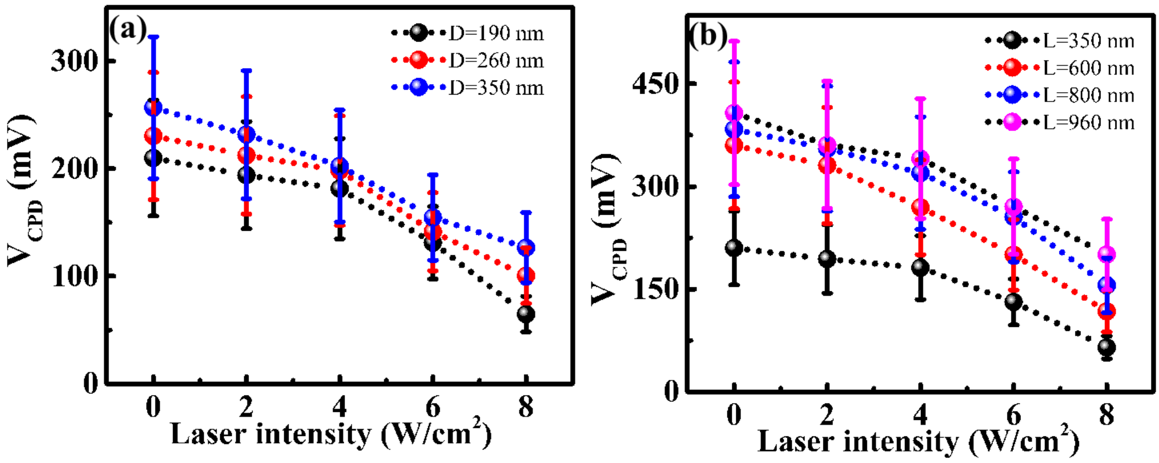

Fig. S5 Contact potential difference (V_{CPD}) obtained from the fitting results as a function of laser intensity on Si NWs with different diameters (a) and different lengths (b).
